# Accuracy and Precision of Model-Based Tracking of a Dynamic Hop Landing Activity

**DOI:** 10.3390/bioengineering12111168

**Published:** 2025-10-28

**Authors:** John D. Holtgrewe, Crystal J. Murray, Dominique A. Barnes, Braden C. Fleming, Jillian E. Beveridge

**Affiliations:** Department of Orthopaedics, Rhode Island Hospital, Warren Alpert Medical School of Brown University, Providence, RI 02903, USA; john_holtgrewe@brown.edu (J.D.H.); crystal_murray@brown.edu (C.J.M.); dominique_barnes@brown.edu (D.A.B.); braden_fleming@brown.edu (B.C.F.)

**Keywords:** biplane videoradiography, anterior cruciate ligament, kinematics, model-based tracking, accuracy

## Abstract

Biplane videoradiography (BVR) is the preferred 3D imaging modality for investigating the relationship between sub-millimeter knee kinematic abnormalities and posttraumatic osteoarthritis risk following anterior cruciate ligament (ACL) injury and surgery. Activity-specific BVR system geometries maximize BVR’s limited field of view which, in turn, influences downstream accuracy. The present work aimed to quantify the accuracy, bias, and precision of the reconstructed 3D tibiofemoral kinematics within a BVR system configured to capture the landing phase of a one-leg hop-for-distance activity. Radio-opaque beads were implanted into the femurs and tibiae of three cadaveric knees to provide the gold-standard kinematics. The specimens were moved through the BVR field of view simulating hop and drop landing motions such that the motion trajectories could better approximate dynamic in vivo velocities. The motions were tracked using both marker- and model-based methods. The mean absolute difference in kinematics between the two tracking methods was used to describe accuracy. Bland–Altman tests were used to quantify bias and precision. Kinematic accuracy ranged from 0.30 to 0.39° for rotations and from 0.34 to 0.50 mm for translations. The magnitudes of absolute difference, bias, and precision were similar regardless of the amount of soft tissue present or velocity of the simulated movement. Our results indicate that our approach for capturing BVR-derived kinematics for a one-leg hop-for-distance is sufficiently accurate to capture the magnitude of differences we expect to observe in a clinical population of ACL-reconstructed patients at long-term follow-up and will be useful to other investigators who may wish to record the hop-for-distance activity using the system geometry and image capture settings described here.

## 1. Introduction

The question of how joint motion abnormalities and posttraumatic osteoarthritis risk following anterior cruciate ligament (ACL) injury and surgery are related remains unanswered. Animal models have provided evidence that joint contact mechanics contribute to cartilage damage [1,2]. Determining whether similar damaging contact mechanics are present in ACL-injured patients is of great interest; however, obtaining these measures clinically requires the measurement of 3D skeletal motion with sub-millimeter accuracy. As recently reviewed by Setliff & Anderst [3], the use of biplane videoradiography (BVR) has been steadily growing across a variety of settings. Nevertheless, a significant constraint of the technology is its small field of view. Thus, optimizing the BVR system’s geometry is task- and joint-specific, and affects downstream measurement accuracy.

Previous work has demonstrated that reconstructed 3D skeletal motion using our BVR system (i.e., model-based tracking) of a single bone with all soft tissues dissected was accurate to at least 0.25 mm for translations and 0.25° for rotations [4]. Although the approach enabled precise control over object velocities via customized mechanical positioning stages, the lack of soft tissues limited the scope of inference of the conclusion. Moreover, the BVR system geometry was optimized to accommodate the positioning stages, resulting in a short source-to-image distance (SID) of ~140 cm compared to the larger SIDs that may be required to capture dynamic lower extremity kinematics. Larger SIDs can lead to greater radiation scatter, which degrades image quality and therefore has relevance to model-based tracking accuracy.

To this end, others have demonstrated rotational and translational accuracies of 0.3–0.9° and 0.3–0.7 mm, respectively, for model-based tibiofemoral tracking using system geometries and parameters tailored to treadmill running [5], overground walking [6], and forward lunges [7]. Accuracies were determined via the mean difference between model-based and marker-based tracking methods. While SIDs were not reported in these studies, separation angles—the angle of the intersecting X-ray beams at the center of the field of view—ranged from 45 to 60° and reflect the task-specific nature of BVR system geometry. Selecting an appropriate angle is a balance between optimizing the collection of in-plane versus out-of-plane motion (relative to the image intensifiers of the BVR system), and image occlusion from the contralateral leg. The BVR system geometries and activity direction in previous works were not optimized for capturing the landing phase of a one-leg hop-for-distance activity—an activity of interest because of its widespread use in clinical rehabilitation programs to gauge functional recovery after ACL reconstruction (ACLR) [8]. The accuracy of model-based tracking using a BVR system geometry optimized for this use has not been fully characterized.

In addition to the single-bone approach mentioned previously, a variety of in vivo and ex vivo approaches have been employed to establish BVR system accuracy. Anderst et al. had the opportunity to work with patients who had radio-opaque beads implanted during surgery [5], providing the ideal scenario to use marker-based kinematics as the ground truth as they have been reported to yield highly precise kinematics—on the order of <0.1 mm [9]. However, the need to surgically implant beads significantly limits the feasibility of in vivo approaches of establishing BVR accuracy and precision, necessitating alternative, cadaveric, approaches. To this end, BVR accuracy studies that have used cadaveric specimens to validate model-based tracking [6,7] have demonstrated accuracies comparable to those reported by Anderst et al. This outcome suggests that the cadaveric approach sufficiently recapitulates in vivo conditions, yielding valid conclusions when the ex vivo experimental conditions are thoughtfully considered.

Additionally, many of the existing studies that have characterized model-based tracking of tibiofemoral kinematics [5,6,7] employed proprietary software and tracking algorithms, limiting the applicability of their reported outcomes to those research groups using the same proprietary resources. The present work circumvents these limitations by using an easily accessible, open-source tracking software (SlicerAutoscoper^M^) [10].

Collectively, the differences in BVR system geometry, functional activity, and tracking algorithm all point to the need to define task- and system-specific BVR approach accuracy. Given our interest in ACL injury and surgery, we propose that capturing the landing phase of a one-leg hop-for-distance activity will offer insight into the interplay between neuromuscular control and knee kinematics as this activity is used routinely in clinical rehabilitation programs to gauge functional recovery after ACL reconstruction (ACLR) [8]. Thus, the aim of the present work was to quantify the accuracy, bias, and precision of model-based tracking of three cadavers with varying degrees of soft tissue volume using a BVR system geometry optimized for the landing phase of the hop-for-distance task specifically. Further, we implement openly available software packages for kinematics reconstruction [9,10] to enhance accessibility and reproducibility in future implementations of our approach.

## 2. Methods

### 2.1. Specimen Preparation

**Figure 1:**
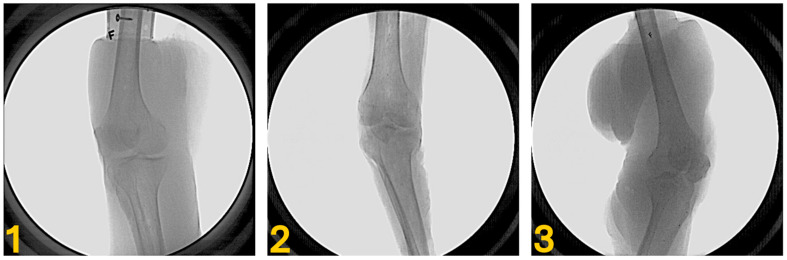
Exemplar BVR images of each specimen (**1**–**3**) demonstrating the range of soft tissue volume. The “F” visible in the images of Specimens **1** and **3** is a radiopaque marker placed on the image intensifier during data collection to identify which of the two image intensifiers of the BVR system captured the radiographs.

Cadaveric knee specimens were obtained from three donors: a 76-year-old male (Specimen 1); a donor of unknown age/sex (Specimen 2); and a 68-year-old female (Specimen 3). These cadaveric specimens were used solely for scientific purposes under ethical approval. The specimens varied in size and in the amount of soft tissue surrounding the knee, i.e., “soft tissue volume” (Figure 1). Based on CT imaging (see Section 2.2), the soft tissue volume for each specimen was 3038 cm^3^ for Specimen 1, 1203 cm^3^ for Specimen 2, and 3964 cm^3^ for Specimen 3. For all specimens, the proximal end of the femur was dissected free of soft tissue and potted in a PVC pipe with resin (Smooth-Cast, Smooth-On, Macungie, PA) to accommodate a fixture that allowed the specimen to be manipulated in the BVR field of view. Between six and eight 1 mm diameter tantalum beads were then implanted in each bone via small incisions to preserve the soft tissue envelope. Shallow pilot holes were drilled, and the beads were press-fitted into the cortical bone. Incisions were sutured closed. Specimens were then frozen in a slightly flexed knee position that approximated the ranges previously observed in vivo during the hop landing task (~20–55°) [11].

### 2.2. Bone Model and Volume Generation

Specimens were imaged using a GE Lightspeed 16 computed tomography (CT) scanner. Scans were taken at 80 kVp with the Smart mA kernel and a 512 × 512 pixel matrix size [4,12]. The average voxel size (±SD) across each scan was approximately 0.49 × 0.49 × 0.625 mm (±0.005 mm in-plane resolution). Beads were segmented (Mimics 23.0, Materialise, Plymouth, MI, USA) from CT images to identify the 3D location of each bead within the CT global coordinate system. Soft tissue volume was determined by segmenting the entire specimen in the CT scan and subtracting the volume of the bones (femur, tibia, fibula, and patella) from the total volume.

The SlicerAutoscoper^M^ (SAM) (v24c43b6; accessed 4 April 2024) was used to generate bone volumes from the CT images required for the model-based tracking. Three-dimensional solid models of the femur and tibia segmentations were also exported from Slicer (Slicer 5.6.1; accessed 4 March 2024) and used to define femoral and tibial anatomical coordinate systems as described previously [13].

### 2.3. BVR System Geometry and Recording Parameters

**Figure 2:**
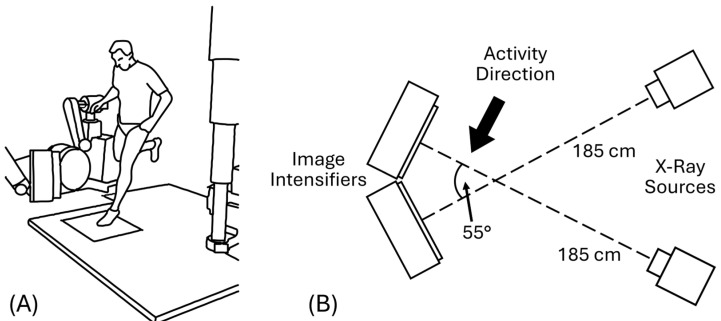
(**A**) BVR system diagram with human subject performing one-leg hop-for-distance; (**B**) top-down diagram showing system geometry and activity direction.

Aspects of the BVR system have been described in detail previously [4] with the exception of the newer IO Industries Flare high-speed digital video cameras (London, ON, Canada). All imaging used an SID of approximately 185 cm and a separation angle of 55° between the two X-ray image intensifiers (Figure 2). This system geometry was optimized to limit occlusion from the contralateral limb while maximizing the likelihood that the knee would be aligned sagittally with one of the image intensifiers to enhance our ability to quantify in-plane anterior tibial translation. X-ray generators were operated in continuous mode with a voltage range of 76–86 kV and a current range of 160–200 mA, while cameras were shuttered at 250 Hz with a 500 µs pulse width to minimize motion blur.

### 2.4. Data Collection

Specimens were kept frozen to ensure that the soft tissues remained evenly distributed over the knee and to provide an additional measurement of kinematic precision such that any non-zero relative tibiofemoral joint motion could be attributed to tracking error [14]. Two motions were recorded: (1) a primarily horizontal “hop” motion that closely simulated a 1-leg hop; and (2) a vertical “drop” to approximate higher velocities recorded during previous in vivo testing (~2.4 m/s) [11]. During data collection, each specimen was fixed to the end of a rod by placing the rod through a hole drilled in the PVC pipe in which the specimen was potted. The other end of the rod was held manually to manipulate the specimen through the BVR field of view while the operator remained shielded from ionizing radiation. For the horizontal “hop” motion, the specimen was held so that the knee joint line was aligned vertically with the centerline of the X-ray emitters and pushed forward into the field of view, mimicking the direction of travel during the in vivo 1-leg hop-for-distance activity shown in Figure 2. For the vertical “drop” motion, specimens were held above the BVR field of view and guided in a near free-fall vertical motion through the field of view until the distal tibia contacted the ground. Three to five trials of each motion were collected during each data collection session. Data collection was repeated for one specimen during a separate imaging session, yielding a total of 33 trials and 1974 trackable frames available for analyses.

### 2.5. Marker-Based Tracking

BVR system calibration and marker-based tracking were conducted using XMALab (XMALab 2.1.0; accessed 10 June 2024) [15]. This approach provides transforms from the CT coordinate system to the BVR global coordinate system for each frame of data with a reported accuracy of 0.01 ± 0.04 mm [9], and was therefore considered the gold standard.

### 2.6. Model-Based Tracking

**Figure 3:**
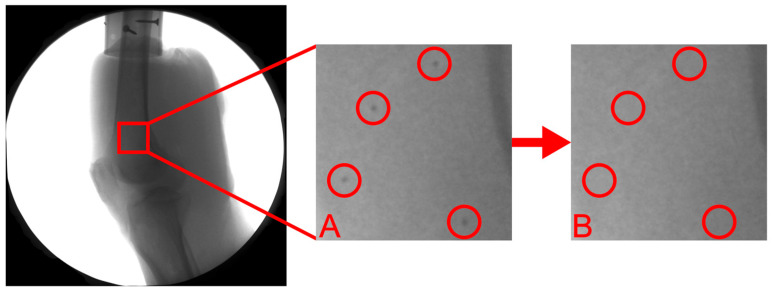
Example of bead masking: (**A**) region of BVR radiograph with beads visible, circled in red; (**B**) identical region after the bead masking algorithm was applied. NB: The image contrast of (**A**,**B**) was enhanced to improve visibility of beads for demonstrative purposes only.

X-ray videos and 3D bone models were processed using custom written software [16] to mask the beads such that their use as additional fiducial markers was eliminated (Figure 3). Model-based tracking was completed using SAM to register the bone volumes generated from the CT scans to the 2D bone motion from the radiographs. SAM [10] is an extension for 3D Slicer [17] that implements Autoscoper, a previously validated open-source 2D-3D registration software program [4,18].

### 2.7. Three-Dimensional Kinematics

Rigid body motions obtained from both tracking methods were converted to quaternions, filtered using a 2nd-order recursive Butterworth filter with a 10 Hz cutoff frequency, and then reconverted to transformation matrices. Six-degree-of-freedom (DOF) Euler angles were resolved in an X-Y-Z sequence, corresponding to flexion/extension (FE), abduction/adduction (AA), internal/external rotation (IE), medial/lateral translation (ML), anterior/posterior translation (AP), and inferior/superior translation (IS) and expressed as the tibial motion relative to the femur using the anatomical coordinate systems derived from the solid bone models [13]. The linear velocity of the tibia for each trial was calculated from the rigid body motions.

### 2.8. Accuracy, Bias, and Precision

Since the specimen was frozen, any change in relative tibiofemoral position can be attributed to tracking error. The mean absolute difference (±SD) between marker- and model-based kinematics was used to describe the tracking accuracy for each DOF. Bland–Altman tests were used to quantify bias and precision, where bias was defined as the mean difference between tracking methods and precision was determined by the limits of agreement (LoA) from the Bland–Altman tests (bias ± 1.96 SD). Four analyses were conducted: (1) with all trial data pooled; (2) femur and tibia analyzed separately by determining the bias and precision of the resolved transforms between CT and BVR coordinate systems for each bone independently; (3) data separated by specimen to evaluate the effect of soft tissue mass; (4) data separated by motion type (e.g., hop vs. drop) to evaluate the effect of velocity. Confidence intervals (CIs) of the LoA were calculated for the pooled analysis. For plotting the mean absolute difference (±SD) between tracking methods, kinematic data for each trial were normalized to a common number of frames: 75 frames for the longer hop trials and 10 frames for the shorter, high-velocity, drop trials.

## 3. Results

**Figure 4:**
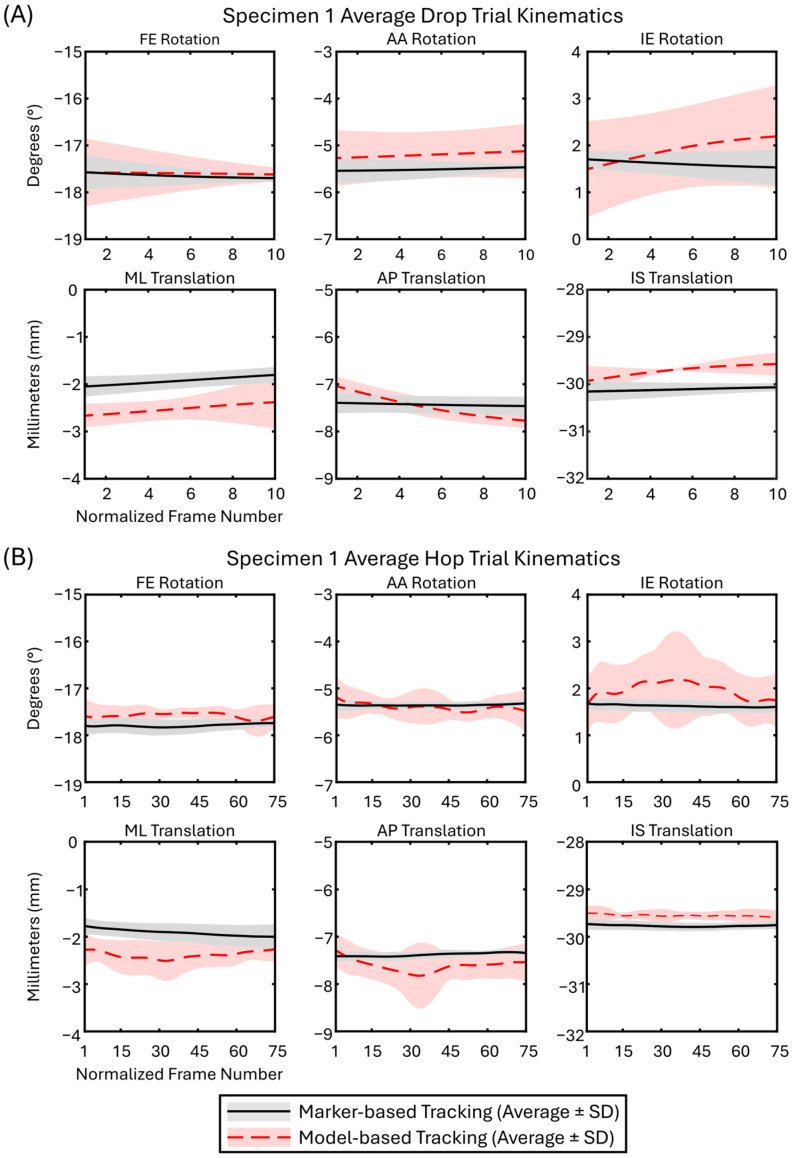
Average 6DOF kinematics across all trials (429 frames of data (27 drop and 402 hop)) for Specimen 1. Based on Bland–Altman analyses, Specimen 1 illustrates the worst-case scenario. (FE = flexion/extension; AA = abduction/adduction; IE = internal/external; ML = medial/lateral; AP = anterior/posterior; IS = inferior/superior). (**A**) Drop trial 6DOF kinematics; (**B**) Hop trial 6DOF kinematics.

For each specimen and each trial, all frames containing the femur and tibia within the field of view were analyzed. Average (±SD) relative kinematics across all trials for Specimen 1 were plotted to provide a sample of the kinematic outcomes from marker-based and model-based tracking (Figure 4). For this sample, the number of frames for each trial was normalized to the average for each trial type: 10 for drop and 75 for hop trials.

**Figure 5:**
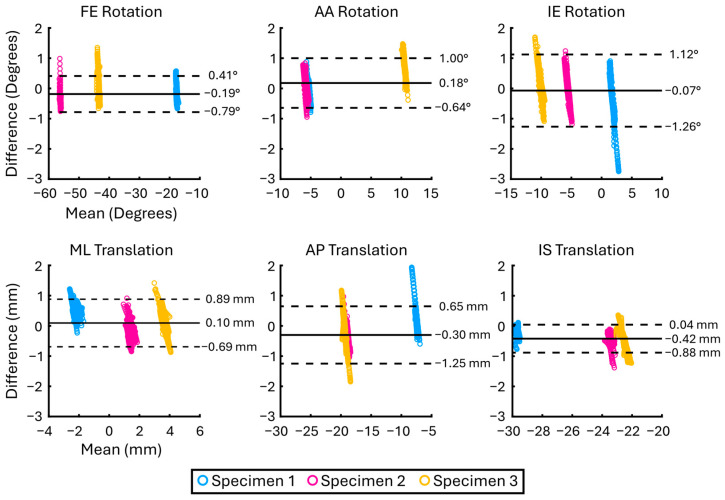
Bland–Altman plots demonstrating the agreement and precision of the model-based tracking kinematics against the gold-standard marker-based kinematics. Limits of agreement were calculated as ±1.96 SD and are noted by the dashed lines. Specimen 2 had the least amount of soft tissue present, while Specimen 3 had the most. The number of data frames for Specimens 1–3 were *n* = 429, *n* = 1095, and *n* = 450, respectively.

When all data were pooled (Table 1), DOFs had similar levels of accuracy (0.3–0.5°/mm) and bias (−0.42–0.10°/mm). For rotational DOFs, LoA CIs were less than 0.1°, and were less than 0.08 mm for translational DOFs. Bland–Altman plots demonstrated the agreement and precision of the model-based tracking for each specimen (Figure 5). The Bland–Altman plots also revealed outliers in the data, particularly for Specimen 1, which had several frames beyond the LoAs in internal/external rotation and anterior/posterior translation DOFs; however, these outlying frames comprised only approximately 5% of the total dataset.

There were no consistent trends in bone-specific accuracy, bias, and precision for the femur and tibia within DOFs (Table 2), as demonstrated by similar mean absolute differences for the femur (0.29°/mm) and tibia (0.21°/mm); however, the femur demonstrated slightly less mean bias (−0.05 vs. 0.18°/mm) and greater mean precision (0.46 vs. 0.69°/mm).

There was no consistent pattern between the amount of soft tissue present and the mean absolute difference and bias in relative tibiofemoral kinematics (Table 3), with the exception of anterior/posterior translation where the width of AP LoAs dovetailed the increase in soft tissue volume: Specimen 2 (0.40 mm) < Specimen 1 (0.80 mm) < Specimen 3 (1.02 mm).

The average velocity of the drop and hop trials was 1.8 ± 0.4 m/s and 0.9 ± 0.3 m/s, respectively. The higher velocity of the drop trials had little effect on the accuracy, bias, and precision of the model-based tracking relative to the marker-based tracking as demonstrated by the nearly even split (55%) in the frequency of accuracy, precision, or bias outcome measures in Table 4 being greater in one trial type versus the other.

**Table 3:** Accuracy, bias, and precision for data analyzed by specimen.

Analysis	DOF	Mean Absolute Difference (±SD) (°/mm)	Bias (°/mm)	LoA (°/mm)
Specimen 1 *n* = 429	FE	0.30 ± 0.17	−0.22	0.52
	AA	0.21 ± 0.16	0.02	0.52
	IE	0.66 ± 0.55	−0.47	1.40
	ML	0.50 ± 0.24	0.49	0.49
	AP	0.39 ± 0.35	0.33	0.80
	IS	0.23 ± 0.13	−0.22	0.27
Specimen 2 *n* = 1095	FE	0.29 ± 0.13	−0.28	0.30
	AA	0.22 ± 0.18	0.02	0.56
	IE	0.40 ± 0.27	−0.04	0.94
	ML	0.26 ± 0.19	−0.09	0.62
	AP	0.53 ± 0.18	−0.52	0.40
	IS	0.42 ± 0.13	−0.42	0.26
Specimen 3 *n* = 450	FE	0.35 ± 0.28	0.07	0.87
	AA	0.72 ± 0.36	0.71	0.72
	IE	0.53 ± 0.33	0.24	1.13
	ML	0.36 ± 0.25	0.17	0.79
	AP	0.53 ± 0.35	−0.36	1.02
	IS	0.62 ± 0.29	−0.60	0.66

**Table 4:** Accuracy, bias, and precision for data analyzed by motion.

Analysis	DOF	Mean Absolute Difference (±SD) (°/mm)	Bias (°/mm)	LoA (°/mm)
Drop (1.8 m/s) *n* = 173	FE	0.40 ± 0.22	0.01	0.90
	AA	0.68 ± 0.39	0.41	1.31
	IE	0.51 ± 0.34	−0.02	1.21
	ML	0.30 ± 0.24	0.16	0.68
	AP	0.37 ± 0.23	0.25	0.69
	IS	0.55 ± 0.30	−0.54	0.63
Hop (0.9 m/s) *n* = 1801	FE	0.30 ± 0.18	−0.21	0.55
	AA	0.30 ± 0.28	0.16	0.74
	IE	0.48 ± 0.38	−0.08	1.19
	ML	0.34 ± 0.24	0.09	0.80
	AP	0.51 ± 0.28	0.31	0.97
	IS	0.41 ± 0.21	−0.41	0.44

## 4. Discussion

Our pooled results showed reasonable agreement with previous measures of idealized conditions without soft tissue present using our BVR system, whereby accuracies of the present analyses ranged from 0.30 to 0.39° for rotations and from 0.34 to 0.50 mm for translations [4]. These magnitudes were only slightly greater than the 0.30° and 0.25 mm rotational and translational accuracies for a single femur [4]. Notably, our pooled results encompass the compound error associated with the relative tibiofemoral kinematics and their soft tissue envelope and compare favorably to the accuracy reported for in vivo tibiofemoral kinematics during downhill running with dynamic rotational and translational accuracies that have been reported to range from 0.3 to 0.9° and from 0.3 to 0.7 mm, respectively [5]. Our results also fall within the range of existing validation values derived from a variety of other in vivo and in situ activities, with reported rotational and translational accuracies of 0.16–0.9° and 0.24–0.78 mm, respectively [3]. It is worth noting that these comparative values represent different outcome measures of accuracy and precision. For example, “accuracy” has been reported as bias and precision [5], root mean squared error [6], and the mean absolute difference [7]. This variation in the numerical derivation of accuracy outcome measures makes direct comparisons across studies difficult. Nevertheless, all approaches—including those presented here—describe similar sub-millimeter accuracy and/or precision during both in vivo and in vitro conditions [5,6,7].

The magnitudes of absolute differences, bias, and LoAs across specimens were similar, regardless of the amount of soft tissue present. Specimens 1 and 3 tended to have larger LoAs; however, this was not consistent across all degrees of freedom and there was no clear trend in the relationship between level of soft tissue volume and mean absolute difference or bias. For example, Specimens 1 and 3 had a lower mean absolute difference or bias compared to Specimen 2, despite having more than double the amount of soft tissue. These results lead us to believe that, on average, we would expect a similar level of accuracy, bias, and precision across varying levels of soft tissue mass in vivo. Nevertheless, the Bland–Altman plots in Figure 5 revealed that there were tracked frames that had a noticeably greater bias relative to the mean bias, particularly for Specimens 2 and 3 in the AP DOF, dovetailing the increasingly wide AP LoAs in these same subjects who coincidentally had an increasing amount of soft tissue. Because the amount of soft tissue was fixed in a frozen state and did not have a systematic effect across all DOFs, we do not believe soft tissue volume is a concern so long as BVR image settings are tuned to maximize cortical bone contrast at the time of image capture. Nonetheless, we acknowledge that it is likely that true biological properties such as tissue elasticity and muscle interaction would not be recapitulated by our approach. Further inspection of the data in question in Figure 5 revealed that the bias in these frames can most likely be attributed to one or both bones being partially out of the field of view and represent only a small proportion of the total number of frames (~5%). In general, frames where bones are not fully in the field of view occur towards the beginning and the end of a trial, or as the bones are coming into or exiting the field of view. Our conclusion from this observation is that users should be conservative in selecting the number of trackable frames as the bones enter the BVR field of view. Furthermore, the bone-specific comparisons in Table 2 showed that the femur and tibia were both tracked with similar levels of accuracy and precision, indicating that our reported tracking error was not overtly biased towards a specific bone. The slightly more favorable femoral tracking is likely explained by the distinctive geometric features afforded by the femoral condyles compared to the more cylindrical tibia. However, tibial tracking accuracy, bias, and precision are still less than those of the relative tibiofemoral kinematics, suggesting that the tibia is not the sole driver of the overall tracking error.

Bone velocity had little effect on the accuracy, bias, and precision, with the faster “drop” trials having similar bias and LoAs to the slower “hop” trials. Again, a small proportion of the frames with the specimen entering the field of view widened some LoAs. The AP biases for the drop trials reported in Table 1 are larger than those described by Giphart et al., who similarly “dropped” their specimen through a BVR system field of view and reported a bias of ~0.1 mm and a mean standard deviation of 0.64 mm [19]. Differences in the number of data frames investigated (27 per subject vs. ~650 analyzed here), idealized versus real-world BVR system geometry, bead visibility in the model-based tracking, averaging of subject biases, and our use of the more conservative LoA precision measure likely contribute to the disagreement in outcomes between the two studies.

Despite the difference in bias magnitudes, the mean sub-millimeter accuracy (<0.4°/mm) and bias (<0.2°/mm) reported here are nearly an order of magnitude smaller than the approximately 3 mm side-to-side differences in arthrometer measures taken in ACL reconstruction patients [20,21,22] and passive tibiofemoral alignment [23] reported by others. Further, Anderst and colleagues have described an increase in dynamic anterior tibial position of approximately 0.9 mm within the first year [5], which could conceivably continue to increase with ACL graft deterioration over time [24]. Given our interest in determining how abnormal joint motion may contribute to posttraumatic osteoarthritis risk at long-term follow-up, the results here provide high confidence that our BVR kinematic outcome measures are sufficiently accurate to capture potentially clinically meaningful long-term in vivo kinematic and alignment changes in ACLR patients.

Although our approach to quantify model-based tracking attempted to simulate in vivo conditions, the true dynamic nature of an in vivo hop landing was not reproduced exactly and is a limitation of this work. Nevertheless, our accuracy is comparable to the accuracy obtained for in vivo running at 2.5 m/s that similarly employed marker-based kinematics as the gold-standard comparator to characterize model-based tracking accuracy [5]. We also chose to use frozen cadaveric specimens to ensure that any relative tibiofemoral joint motion could be attributed to tracking error, which is minimal and can be appreciated from Figure 1. A strength of our analyses, which has not been described by others, was that the bead masking algorithm allowed us to eliminate any supplementary fiduciary information they could have provided during model-based tracking. This strength is notable in the context of the older cadaveric specimens with lower cortical bone quality, which represents a worst-case scenario for model-based tracking. Younger subjects (<40 years old) with robust, cortical bone would provide greater image contrast to enhance the pixel intensity-based model-based tracking method employed by SAM. Further to this point, the average Hounsfield units of the femurs and tibias of the specimens employed in this study were approximately 30% lower than the younger ACLR population we have studied to date [12,22,25]. Thus, there is reason to believe that the model-based tracking errors presented here are conservative and it is plausible that tracking could improve with younger participants. Lastly, the donor age and sex were unknown for Specimen 2; however, given it was obtained through the same donor program as the other specimens which has an average donor age of approximately 65, we believe it is highly likely to be consistent. A post hoc analysis of cortical bone Hounsfield units supports this assumption, with Specimen 2 having an average HU value of 499.6 compared to 469.7 and 551.2 of Specimens 1 and 3, respectively. For these reasons we do not believe the data from Specimen 2 of unknown age and sex introduce undue bias in our analyses and are unlikely to have affected our results.

## 5. Conclusions

In conclusion, the accuracy, bias, and precision of tibiofemoral joint motion during a hop landing captured by our tailored BVR configuration and post-processing approach are sufficiently sensitive to capture clinically meaningful long-term functional changes in our patient population of interest. In future work, we plan to implement the BVR system geometry and post-processing markerless tracking approach described here to characterize kinematic abnormalities following ACL injury and surgery while continuing to contribute to the development, optimization, and deployment of SAM [10].

## Figures and Tables

**Table 1:** Pooled accuracy, bias, and precision between marker- and model-based tracking. A total of *n* = 1974 frames of data were analyzed.

DOF	Mean Absolute Difference (±SD) (°/mm)	Bias (°/mm)	LoA (°/mm)
FE	0.30 ± 0.19	−0.19	0.60
AA	0.33 ± 0.31	0.18	0.82
IE	0.49 ± 0.37	−0.07	1.19
ML	0.34 ± 0.24	0.10	0.79
AP	0.50 ± 0.28	−0.30	0.95
IS	0.43 ± 0.23	−0.42	0.46

**Table 2:** Accuracy, bias, and precision for femur and tibia analyzed separately. The number of data frames included in each analysis is noted in the lefthand column.

Analysis	DOF	Mean Absolute Difference (±SD) (°/mm)	Bias (°/mm)	LoA (°/mm)
Femur *n* = 1974	FE	0.21 ± 0.14	−0.14	0.42
	AA	0.17 ± 0.11	−0.02	0.40
	IE	0.31 ± 0.22	−0.10	0.72
	ML	0.37 ± 0.29	0.32	0.67
	AP	0.20 ± 0.12	−0.15	0.36
	IS	0.49 ± 0.10	−0.49	0.20
Tibia *n* = 1974	FE	0.17 ± 0.15	0.05	0.44
	AA	0.26 ± 0.23	−0.13	0.64
	IE	0.42 ± 0.34	−0.02	1.06
	ML	0.48 ± 0.25	0.19	1.00
	AP	0.24 ± 0.16	0.06	0.56
	IS	0.18 ± 0.13	0.03	0.43

## Data Availability

The raw data supporting the conclusions of this article will be made available by the authors on request.

## Abbreviations

The following abbreviations are used in this manuscript:

BVR	Biplane Videoradiography
ACL	Anterior Cruciate Ligament
SID	Source-to-image distance
ACLR	ACL Reconstruction
SAM	SlicerAutoscoper^M^
DOF	Degree-of-freedom
FE	Flexion/Extension
AA	Abduction/Adduction
IE	Internal/External
ML	Medial/Lateral
AP	Anterior/Posterior
IS	Inferior/Superior
LoA	Limits of Agreement
CI	Confidence Intervals
HU	Hounsfield Units
